# The effect of mobile application interventions on influencing healthy maternal behaviour and improving perinatal health outcomes: a systematic review protocol

**DOI:** 10.1186/s13643-017-0424-8

**Published:** 2017-02-08

**Authors:** Lisa M. Daly, Dell Horey, Philippa F. Middleton, Frances M. Boyle, Vicki Flenady

**Affiliations:** 1 Mater Research Institute, The University of Queensland, South Brisbane, QLD Australia; 2 School of Psychology and Public Health, La Trobe University, Bundoora, VIC Australia; 3 Healthy Mothers, Babies and Children’s Theme, SAHMRI, Adelaide, SA Australia; 4 School of Public Health, The University of Queensland, Herston, QLD Australia

**Keywords:** Apps, Pregnancy, Perinatal, Maternal, Infant, Mobile, Systematic review, Behaviour change, Intervention

## Abstract

**Background:**

Perinatal morbidity and mortality remain significant public health issues globally, with enduring impact on the health and well-being of women and their families. Pregnant women who adopt, practice and maintain healthy behaviours can potentially improve the health of themselves and their babies. Mobile applications are an increasingly popular mode of accessing, storing and sharing health information among pregnant women. The main objective of this review is to evaluate the effects of mobile application interventions during pregnancy on maternal behaviour and associated maternal and infant outcomes.

**Methods:**

This review will include randomised and non-randomised studies which tested use of mobile applications designed to improve either maternal knowledge or behaviours to address known risk factors associated with adverse perinatal health outcomes. This review will include studies which included pregnant women and/or women during birth. The search strategy will utilise a combination of keywords and MeSH terms. Literature databases such as PubMed, Embase, The Cochrane Library, CINAHL and WHO Global Health Library will be searched. Two reviewers will independently screen retrieved citations to determine if they meet inclusion criteria. Studies will be selected that provide information about interventions commenced in early pregnancy, late pregnancy or labour.

Comparisons to be made include mobile applications versus interventions relying on paper-based or text-messaging-based communication; interpersonal communication such as face-to-face or telephone conversation; and no intervention or standard care. Quality assessment of included randomised studies will utilise established guidelines provided in the *Cochrane Handbook for Systematic Reviews of Interventions*. Quality assessment of non-randomised studies will be based on the *Risk of Bias in Non-randomised Studies-of Interventions* (*ROBINS-I*) assessment tool. Quality of the evidence will be evaluated using the *Grades of Recommendation, Assessment, Development and Evaluation* (*GRADE*) approach. Separate comparisons and analyses for primary and secondary outcomes will be performed. Results of the review will be reported according to the *Preferred Reporting Items for Systematic Reviews and Meta-Analyses* (*PRISMA*) guidelines.

**Discussion:**

This systematic review will identify and synthesize evidence about the effect of interventions delivered through mobile applications on influencing maternal behaviour and improving perinatal health outcomes.

**Systematic review registration:**

PROSPERO CRD42016037344.

**Electronic supplementary material:**

The online version of this article (doi:10.1186/s13643-017-0424-8) contains supplementary material, which is available to authorized users.

## Background

Perinatal morbidity and mortality remain significant public health issues globally, with enduring impact on the health and well-being of women and their families. Pregnant women who adopt, practice and maintain healthy behaviours can potentially improve the health of themselves and their babies. Some maternal risk factors for adverse perinatal health outcomes, such as obesity, smoking, substance use, hypertension, diabetes, adequate nutrition [1–3] and maternal perception of decreased fetal movement [4], may be modifiable through changes in maternal behaviour. These risk factors also have an association with increased rates of stillbirth, pre-term birth, low birthweight and small for gestational age babies, and emergency caesarean section [5].

Health communication methods such as face-to-face education, pamphlets, audio-visual training clips and mass media have been employed for decades to encourage healthy behaviours among pregnant women. However, women are increasingly turning to digital sources of information during pregnancy [6], and may prefer these modalities over traditional, paper-based formats [7].

The emergence of mobile health modalities extends the opportunity to reach, teach, connect, motivate, and empower individuals to address specific health concerns [8]. Mobile phone-based interventions represent a shift in health communication modalities to a dynamic, interactive environment that can include verbal, vocal, and visual messages [9]. These interventions provide individual-level support to pregnant women, due to their popularity, mobility, technological capabilities, and availability [10]. Broadly, mobile health strategies have the potential to improve perinatal outcomes by improving access to health information, modifying demand for quality services, and enabling the provision of targeted care [11].

In particular, mobile applications may assist women with tools to support healthier lifestyles and strengthen informed decision-making. Mobile applications (“apps”) are computer programs designed to run on mobile devices such as smartphones and tablet computers. Apps became widely available for consumer download from 2008, with the launch of “app marketplaces” tailored to the user’s mobile operating system [12]. There are now over 100,000 health and medical apps available for use by lay people and healthcare workers, and apps directed at pregnancy constitute a major genre [13, 14]. These applications can include health information, motivational messages, monitoring, and behaviour change tools, with content tailored by demographics such as maternal age, gestational age, health issues or other known risk factors, cultural affiliation, or language. Apps have become a popular modality to access, store, and share health information.

Recent studies have found that pregnant women are seeking mobile apps to provide information, to monitor fetal development and changes in their own bodies and to provide reassurance [6, 15–17]. Pregnant women may also feel heightened support for informed decision-making and sense of control, using a familiar device to access, store and share information. One Australian study found that 75% of pregnant women had downloaded at least one pregnancy app, and the majority of these women used one of these apps at least once per week [15].

The number of pregnant women and women of childbearing age already using pregnancy-related mobile applications to access information, track individual health indicators and assist in decision-making is rising exponentially. Collectively, pregnancy apps available through app stores GooglePlay and iTunes have been downloaded hundreds of millions of times and are an integral source of information for many pregnant women, particularly in high-income countries [18].

Health systems and maternity care facilities are questioning whether and how to integrate these patient support modalities to improve outcomes for mothers and newborns. However, limited evidence exists to support the value of mobile applications, compared to other communication methods, in terms of maternal behaviour change or perinatal health outcomes. Similarly, few studies have reviewed the content delivered to pregnant women through mobile applications, and whether content is accurate, addresses specific need of women with high-risk pregnancies or pre-existing medical conditions [19].

Mobile phone-based applications offer a new frontier to test theories of behaviour change, health promotion and health care-seeking behaviour. To date, most published studies of mobile phone interventions have analysed text messaging as the primary mode of health communication [20, 21], as opposed to an interactive mobile application. Investigation of the effectiveness of mobile applications for provision of perinatal health information, and their impact on maternal behaviour and associated maternal and infant outcomes, is particularly topical.

### Aim

This review aims to assess the effects of mobile application interventions during pregnancy on influencing healthy maternal behaviour and improving perinatal health outcomes, compared with interventions using other communication modalities or with standard care.

## Methods

### Protocol

This systematic review protocol has been developed based on Preferred Reporting Items for Systematic Reviews and Meta-Analysis Protocols (PRISMA-P) guidelines for reporting systematic reviews evaluating health care interventions [22, 23]. A PRISMA-P checklist file is attached (Additional file 1).

### Eligibility criteria

Studies will be selected according to the criteria outlined below.

### Participants

Included studies will involve women during any stage of pregnancy, or labour.

### Interventions

Studies assessing the effects of mobile application-based interventions designed to influence maternal knowledge or behaviour during pregnancy will be considered for inclusion. Mobile application-based interventions will be included if they provide general information for pregnant women, or focus on a specific maternal risk factor or perinatal outcome. There will be no restrictions on who has sponsored the intervention; for example, mobile apps developed commercially will be eligible, as will mobile apps developed by hospitals, health systems or other organisations.

Studies will be *excluded* if they fulfil any of the following criteria:

- ▪ Do not utilise a mobile application
- ▪ Mobile phone is used solely for telephone conversations or text messaging
- ▪ Do not report on a maternal or infant health outcome
- ▪ Do not describe mobile application interventions for pregnant women (as opposed to health care workers, clinicians, partners, etc.)
- ▪ Study focuses on physical effects of mobile phone usage (such as radiation) during pregnancy

### Comparators

The following comparisons will be made:

1. Mobile health application versus paper-based or text-messaging-based communications
2. Mobile health application versus interpersonal communication modes (face-to-face or telephone conversation)
3. Mobile health application versus no specified intervention, or standard care.

### Outcomes

- Primary outcomes
  Change in maternal behaviours (as defined by trial authors), by intervention goals; for example, adoption of healthier lifestyles, smoking cessation, increased physical activity, weight control, glucose control, improved nutrition, timely reporting of pregnancy concerns).
- Secondary outcomes
  - Maternal outcomes
    - ▪ Major adverse maternal outcome (composite of death, admission to intensive care unit or near-miss mortality as defined by World Health Organization (WHO)).
    - ▪ Antepartum haemorrhage
    - ▪ Postpartum haemorrhage
    - ▪ Pre-eclampsia
    - ▪ Gestational diabetes mellitus (GDM)
    - ▪ Emergency caesarean birth
    - ▪ Successful initiation of breastfeeding
    - ▪ Maternal knowledge (about the health topic (s) that is/are the target of the intervention)
    - ▪ Maternal general health (as defined by standardised measures such as general health questionnaires)
    - ▪ Maternal evaluation of the intervention (as reported by the trial)
    - ▪ Maternal psychosocial outcomes, such as satisfaction, self-efficacy or anxiety (as measured by any validated, standard instrument)
    - ▪ Maternal-infant bonding (as reported by the trial)
    - ▪ Maternal perception of communication with health care providers
    - ▪ Health service utilisation (antenatal care attendance, maternal antenatal admission, length of hospital stay of mother or infant)
  - Infant outcomes
    - ▪ Stillbirth
    - ▪ Neonatal death
    - ▪ Small for gestational age (SGA)
    - ▪ Large for gestational age (LGA)
    - ▪ Preterm birth (before 32 weeks)
    - ▪ Gestational age at birth
    - ▪ Caesarean section
    - ▪ Neonatal intensive care unit (NICU) admissions
    - ▪ Infant growth/weight gain
    - ▪ Major neonatal morbidities (as defined by the trial authors)

For any outcomes reported as a composite measure, we will extract all composite and individual outcomes as reported in the studies, where available.

### Setting

There will be no restrictions by type of setting.

### Time frame

Studies will be selected that provide information about interventions commenced during pregnancy and/or during birth. Measurement of outcomes will focus on the perinatal period as defined by the Australian Institute of Health and Welfare, as 20 weeks of pregnancy to 28 days after birth [24].

### Study designs

We will include randomised controlled trials (RCTs) (including cluster RCTs) and non-randomised studies including controlled before-and-after studies, interrupted time-series studies and prospective comparative cohort studies. Case-control studies, crossover trials and cross-sectional studies will be excluded.

## Information sources

We will perform a systematic literature search using the following electronic bibliographic databases: PubMed, Embase, The Cochrane Library, CINAHL and WHO Global Health Library. Collections such as POPLINE database and CABI Global Health will also be searched. We will conduct handsearches of journals or conference proceedings from the reference lists of retrieved studies. No language or date restrictions will be applied. Abstracts and full-length articles will be obtained for each citation. Reference lists of included studies and relevant reviews will be scanned to ensure coverage of the literature. The search will only include peer-reviewed publications.

## Search strategy

The specific search strategies were developed by the primary author and an experienced clinical research librarian, with input from all authors. An electronic search using subject headings and all fields for keywords will be conducted to avoid missing non-indexed concepts. Literature searches will be re-run just before final analyses and further studies retrieved for inclusion. Search terms and search strategy example are provided in Additional file 2.

## Study records

### Data management

Reference management software EndNote will be used to organise articles describing studies identified in the literature search. Search results from different electronic databases will be combined in a single EndNote library and duplicate records of the same reports will be removed.

Remaining literature search results will be uploaded to Covidence [25], a web-based software platform that supports citation screening and facilitates collaboration among multiple authors. The team will develop and test screening questions and forms based on the review inclusion and exclusion criteria. Citation abstracts and full text articles will be uploaded to the Covidence software.

### Selection process

Two reviewers will independently screen the title and abstract of retrieved citations to determine if they meet inclusion and exclusion criteria, using the Covidence software. If there is a concern about inclusion, the abstract will be reviewed by a third author to determine if it meets the inclusion criteria. If necessary, study authors will be contacted up to two times to resolve questions about eligibility. Reasons will be recorded for excluding trials.

Studies will be included only once for evaluation, and multiple publications from the same study will be reported. The publication which most comprehensively describes the results will be used as a primary reference. Review authors will not be blind to the journal titles, study authors or institutions, as several studies have found that such masking is of limited value in study selection for systematic reviews [26, 27]. For selected studies, full articles will be obtained and read by two authors to confirm that they meet inclusion criteria. Any differences of opinion between two reviewers will be resolved by a third review author and consensus. A Preferred Reporting Items for Systematic Reviews and Meta-analyses (PRISMA) flow-diagram [28] will summarise the inclusion and exclusion of studies.

### Data collection process

Two authors will independently extract outcome data using standardised forms, once studies are selected. Data extraction will include study objective, study design, inclusion and exclusion criteria, data sources, study period, methodology, population size, intervention details, duration, intensity and effects and outcomes. A standardised form will be designed to extract data required for the quality assessment and evidence synthesis, and summary tables will be populated. Each author will thoroughly review the summary tables for relevance and accuracy. Any differences of opinion between two reviewers will be resolved by a third review author and consensus. If necessary, study authors will be contacted up to two times to provide further details. Data will be entered into Review Manager software [29] and checked for accuracy.

## Assessment of risk of bias

### Quality assessment

Risk of bias for randomised trials will be assessed using established guidelines provided in the Cochrane Handbook for Systematic Reviews of Interventions [30]. Six domains are covered in this guideline: selection bias, performance bias, detection bias, attrition bias, reporting bias and other bias. Explicit judgements will be made about whether studies are at high risk of bias, according to the criteria given in the handbook [30]. The likely magnitude and direction of the bias, and whether it is likely to have impacted findings, will be assessed. The impact of the level of bias identified will be explored through undertaking sensitivity analyses.

Quality assessment of non-randomised studies will be based on the Risk of Bias in Non-randomised Studies-of Interventions (ROBINS-I) assessment tool [31].

Two reviewers will independently assess the risk of bias of included studies. Any disagreements between the review authors over the risk of bias in particular studies will be resolved by discussion, with involvement by a third review author where necessary.

## Assessment of the body of evidence—the GRADE approach

Quality of the evidence will be evaluated using the Grades of Recommendation, Assessment, Development and Evaluation (GRADE) approach [32]. The GRADE approach uses five considerations (study limitations, consistency of effect, imprecision, indirectness and publication bias) to assess the quality of the body of evidence for specific outcomes. The evidence can be downgraded from ‘high quality’ by one level for serious (or by two levels for very serious) limitations, depending on assessments for risk of bias, indirectness of evidence, serious inconsistency, imprecision of effect estimates or potential publication bias.

## “Summary of findings” tables

GRADE Profiler [33] will be used to import data from *Review Manager* software [29] in order to create a “Summary of Findings” table. A summary of intervention effect and a measure of quality according to the GRADE approach [34] will be presented as tables for the following outcomes of interest:

1. Maternal behaviours achieved (primary outcome)
2. Maternal knowledge (about the health topic that is the target of the intervention)
3. Maternal evaluation of the intervention
4. Maternal psychosocial outcomes
5. Health service utilisation
6. Perinatal mortality
7. Major neonatal morbidities

## Measures of treatment effect

Randomised control trials (RCTs) and non-RCTs will be analysed separately. For intervention studies including randomised control trials, results will be presented as summary risk ratios (RR) with 95% confidence intervals (CI) for dichotomous outcomes. For continuous data, the mean difference will be used if outcomes are measured in the same way between trials. The standardised mean difference will be used to combine trials that measure the same outcome, but use different methods.

For cohort studies, effect estimates will be presented where possible as percentages, relative risk (RR) or odds ratios (OR) with 95% CIs, or adjusted RR or OR if reported with 95% CIs, in tabular format based on study type; narrative synthesis will summarise the studies. Random effects meta-analyses will be performed, accounting for variation of diverse studies.

### Unit of analysis issues

Cluster-randomised trials will be included in analyses along with individually randomised trials. Sample sizes will be adjusted using an estimate of the intracluster correlation co-efficient (ICC) derived from the included trial (if possible), from a similar trial or from a study of a similar population. If ICCs are used from other sources, this will be reported and sensitivity analyses conducted to investigate the effect of variation in the ICC. If both cluster-randomised and individually randomised trials are identified, relevant information will be synthesised. It will be considered reasonable to combine the results from both if there is little heterogeneity between the study designs and the interaction between the effect of intervention and the choice of randomisation unit is considered to be unlikely.

Heterogeneity in the randomisation unit will also be acknowledged, and a sensitivity analysis performed to investigate the effects of the randomisation unit.

### Dealing with missing data

For included studies, levels of attrition will be noted. The impact of including studies with high levels of missing data will be explored in the overall assessment of treatment effect by using sensitivity analysis.

For all outcomes, analyses will be carried out, as far as possible, on an intention-to-treat basis, i.e. with attempts to include all participants randomised to each group in the analyses (for RCTs), and all participants analysed in the group to which they were allocated, regardless of whether or not they received the allocated intervention. The denominator for each outcome in each RCT will be the number randomised minus any participants whose outcomes are known to be missing.

### Assessment of heterogeneity

Heterogeneity between studies will be assessed by comparison of study settings, populations and design, supplemented with the I^2^ statistic. Where data is not presented in such a way that inclusion in meta-analysis is possible, or where only one study is identified for a risk factor, results of individual studies will be presented. Statistical heterogeneity will be assessed in each meta-analysis using the T^2^, I^2^ and Chi^2^ statistics. We will regard heterogeneity as substantial if I^2^ is greater than 30% and either T^2^ is greater than zero or there is a low *P* value (<0.10) in the Chi^2^ test for heterogeneity.

### Assessment of reporting biases

If there are 10 or more studies in the meta-analysis, reporting biases (such as publication bias) will be investigated using funnel plots. Funnel plot asymmetry will be assessed visually. If asymmetry is suggested by a visual assessment, exploratory analyses will be performed to investigate further.

### Data synthesis

Statistical analyses will be carried out using the Review Manager (RevMan) software [29]. Fixed-effect meta-analysis will be used for combining data where it is reasonable to assume that studies are estimating the same underlying treatment effect: i.e. where trials are examining the same intervention, and the trials’ populations and methods are judged sufficiently similar.

If there is clinical heterogeneity sufficient to expect that the underlying treatment effects differ between trials, or if substantial statistical heterogeneity is detected, random-effects meta-analysis will be used to produce an overall summary, if an average treatment effect across trials is considered clinically meaningful. The random-effects summary will be treated as the average range of possible treatment effects and clinical implications of treatment effects differing between trials will be discussed. If the average treatment effect is not clinically meaningful, trials will not be combined.

If random-effects analyses are used, the results will be presented as the average treatment effect with 95% confidence intervals, and the estimates of T^2^ and I^2^. Where studies have used the same type of intervention and comparator, with the same outcome measures, results will be pooled using random-effects meta-analysis, with standardised mean differences for continuous outcomes and risk ratios for binary outcomes. If formal pooling is inappropriate for analysis, data synthesis will employ a narrative and tabular approach.

### Subgroup analysis and investigation of heterogeneity

Subgroup analyses will be performed according to the type of comparator, if studies permit.

Separate comparisons and analyses for primary and secondary outcomes will be performed where possible, and analyses will be performed for the following subgroups, if studies permit:

1. Low-resource versus high-resource settings.
2. Maternal characteristics by age and parity.
3. Approach to information available on-demand used by mobile application (“pull” communication), or timed delivery of information (“push communication”).

If substantial heterogeneity is identified, it will be investigated using subgroup analyses and sensitivity analyses. Authors will consider whether an overall summary is meaningful, and if it is, use random-effects analysis to produce it.

Subgroup differences will be assessed by interaction tests available within RevMan [29]. Results of subgroup analyses will quote the Chi^2^ statistic and *P* value, and the interaction test I^2^ value.

### Sensitivity analysis

Sensitivity analyses will be carried out to explore the effects of high attrition rates with studies showing attrition greater than 20% excluded from the analyses in order to assess whether there are any differences to the overall result. Where ICCs are used, sensitivity analyses will explore the effects of variation in ICC values and in the randomisation unit (i.e. individual versus cluster).

## Discussion

Millions of women, across many countries, utilise mobile applications regularly during pregnancy, for purposes of gathering information, data tracking, information sharing, education and reassurance. This systematic review is the first to assess the effects of mobile application interventions during pregnancy on influencing healthy maternal behaviour and improving perinatal outcomes. Results of this systematic review could contribute to decision-making by health systems, hospitals and clinicians about whether integration of mobile applications might influence the knowledge and behaviour of women in their care, particularly those demonstrating risk factors.

## Additional files

Additional file 1: PRISMA-P checklist. This checklist includes a list of recommended items to include in a systematic review protocol. (DOCX 28 kb)
Additional file 2: Search terms and search strategy. This search strategy tailored for PubMed will be adapted for each database. (DOCX 17 kb)

## Abbreviations

Apps: Mobile applications
CI: Confidence interval
GDM: Gestational diabetes mellitus
GRADE: Grades of recommendation, assessment, development and evaluation
ICC: Intracluster correlation co-efficient
LGA: Large for gestational age
MESH: Medical subject headings
OR: Odds ratio
PRISMA-P: Preferred Reporting Items for Systematic Reviews and Meta-Analysis Protocols
PROSPERO: International Prospective Register of Systematic Reviews
RCT: Randomised controlled trial
ROBINS-I: Risk of bias in non-randomised studies–of interventions assessment tool
RR: Risk ratio
SGA: Small for gestational age
WHO: World Health Organization
