# Supplementary material for: The effect of mobile application interventions on influencing healthy maternal behaviour and improving perinatal health outcomes: a systematic review protocol
Source: Syst Rev. 2017 Feb 8;6:26. doi: 10.1186/s13643-017-0424-8 (PMC5299644; doi:10.1186/s13643-017-0424-8)
Supplement: Additional file 2: — Search terms and search strategy. This search strategy tailored for PubMed will be adapted for each database. (DOCX 17 kb) [file 13643_2017_424_MOESM2_ESM.docx]

**Additional file 2: Search terms and search strategy**

Search terms will be adapted for use with bibliographic databases in combination with database-specific filters for controlled trials, where these are available. The following search terms will be used:

|  |  |
| --- | --- |
| **Participant characteristics** | **Intervention mode** |
| **All fields** | |
| pregnan*  matern*  mother*  prenat*  pre-nat*  perinat*  peri-nat*  antenat*  ante-nat*  reproducti* | "mobile app*"  mobile application (MESH)  “mobile phone”  “cell phone”  smartphone  iPhone  iPad  android  “handheld computers” |
| **MESH** | |
| “Pregnancy”  “Pregnant Women” | “Mobile Applications”  “Smartphone”  “Cell Phones”  “Computers, Handheld” |

An example search strategy for *PubMed* is below. After the search strategy is finalized, it will be adapted to the syntax and subject headings of other databases.

|  |  |  |  |
| --- | --- | --- | --- |
| **Search strategy** | |  |  |
| 1 | pregnan* | 14 | “mobile applications (MESH) |
| 2 | pregnancy (MESH) | 15 | “mobile phone” |
| 3 | “pregnant women” (MESH) | 16 | “cell phone*” |
| 4 | matern* | 17 | “cell phones” (MESH) |
| 5 | mother* | 18 | smartphone |
| 6 | prenat* | 19 | Smartphone (MESH) |
| 7 | pre-nat* | 20 | iPhone* |
| 8 | perinat* | 21 | iPad* |
| 9 | peri-nat* | 22 | android* |
| 10 | antenat* | 23 | “handheld computers” |
| 11 | ante-nat* | 24 | “computers, handheld” (MESH) |
| 12 | reproducti* | 25 | or/1-12 |
| 13 | “mobile app*” | 26 | or/13-24 |
|  |  | 27 | 25 AND 26 |
